# Poor response to neoadjuvant chemotherapy in metaplastic breast carcinoma

**DOI:** 10.1038/s41523-021-00302-z

**Published:** 2021-07-22

**Authors:** Willard Wong, Edi Brogi, Jorge S. Reis-Filho, George Plitas, Mark Robson, Larry Norton, Monica Morrow, Hannah Y. Wen

**Affiliations:** 1 Department of Pathology, Memorial Sloan Kettering Cancer Center, New York, NY USA; 2 Department of Surgery, Memorial Sloan Kettering Cancer Center, New York, NY USA; 3 Department of Medicine, Memorial Sloan Kettering Cancer Center, New York, NY USA

**Keywords:** Breast cancer, Breast cancer

## Abstract

Metaplastic breast carcinoma (MpBC) is a rare special histologic subtype of breast carcinoma characterized by the presence of squamous and/or mesenchymal differentiation. Most MpBCs are of triple-negative phenotype and neoadjuvant chemotherapy (NAC) is frequently utilized in patients with MpBC. The aim of this study was to evaluate response to NAC in a retrospective cohort of MpBCs. We identified 44 patients with MpBC treated with NAC at our center between 2002 and 2018. Median age was 48 years, 86% were clinical stage II–III, and 36% were clinically node-positive. Most (80%) MpBCs were triple-negative or low (1–10%) hormonal receptor positive and HER2 negative on pre-NAC biopsy. While on NAC, 49% showed no clinical response or clinico-radiological progression. Matrix-producing subtype was associated with clinico-radiological response (*p* = 0.0036). Post NAC, two patients initially ineligible for breast-conserving surgery (BCS) were downstaged to be eligible for BCS, whereas three patients potentially eligible for BCS before treatment became ineligible due to disease progression. Only one (2%) patient had a pathologic complete response (pCR). Among the 16 patients presenting with biopsy-proven clinical node-positive disease, 3 (19%) had nodal pCR. Axillary lymph node dissection was avoided in 3 (19%) patients who had successful axillary downstaging. Residual cancer burden (RCB) was assessed in 22 patients and was significantly associated with disease-free survival and overall survival. We observed a poor response or even disease progression on NAC among patients with MpBC, suggesting that NAC should be reserved for patients with inoperable MpBC.

## Introduction

Metaplastic breast carcinoma (MpBC) is a rare special histologic subtype of breast carcinoma, which includes a heterogeneous group of invasive carcinomas characterized by the presence of squamous and/or mesenchymal differentiation^1^. Histologic variants that are recognized by the World Health Organization (WHO) include spindle cell carcinoma, squamous cell carcinoma, and metaplastic carcinoma with mesenchymal differentiation, which includes matrix-producing carcinoma. Low-grade adenosquamous carcinoma and fibromatosis-like metaplastic carcinoma are the rare low-grade variants with a relatively favorable prognosis which differs from the more common high-grade MpBCs^2–5^.

MpBC typically presents as a rapidly growing mass that is often palpable and larger in size compared to invasive carcinoma no special type (NST)^6,7^. MpBCs are usually high grade and negative for estrogen receptor, progesterone receptor, and HER2 (i.e. of triple-negative phenotype)^6,8–10^. Despite the large tumor size at presentation, axillary lymph node involvement is infrequent, and local recurrences and distant metastases are thought to occur by hematogenous dissemination^10,11^. Patients with MpBC tend to have worse disease-free survival and overall survival when compared to common forms of triple-negative breast cancer (TNBC)^10,12–19^.

Given the advanced stage at initial presentation and triple-negative receptor status, neoadjuvant chemotherapy (NAC) is often considered a treatment option for patients with MpBC. Although rates of pathologic complete response (pCR) are relatively high in TNBC NST, at ~30–40% with anthracycline and taxane^20,21^, and over 50% with platinum^22,23^, the response to NAC in MpBC has not been well studied and reported rates of pCR are variable, ranging from 10% to 17%, with sample size ranging from 6 to 29 patients^24–28^. Here we sought to study the response to NAC in patients with MpBC and clinical, radiologic, and pathologic features that were associated with response.

## Results

### Pre-NAC clinicopathological characteristics

From 2002 to 2018, 44 patients with MpBC treated with NAC were identified in the institutional database. The clinicopathologic characteristics of pre-NAC are summarized in Table 1. The median age at diagnosis was 48 years (range: 26–77). Median clinical tumor size was 4.1 cm (range: 2.0–11.9 cm). Forty (91%) patients had clinical T2 or T3 tumors, and 16 (36%) had biopsy-proven node-positive disease at presentation. Twenty-eight (64%) patients were clinical stage II and 10 (23%) were clinical stage III. Among the 31 patients who had genetic testing, 28 (90%) were negative for *BRCA* germline mutations, 2 (6%) were *BRCA1* germline mutation carriers, and 1 (3%) had *BRCA2* variant of unknown significance. The tumor histology was pure metaplastic carcinoma in 17 (39%) patients and mixed metaplastic and invasive carcinoma NST in 27 (61%). Histologic subtypes of metaplastic carcinoma included matrix-producing (19; 43%), squamous cell (12; 27%), spindle cell (6; 14%), with mixed metaplastic elements (6; 14%), and not specified (1; 2%). No low-grade variants of MpBC were included. Tumor-infiltrating lymphocytes (TILs) were assessed in 31 pre-NAC biopsies with available material for review at the time of the study. The majority (65%, 20/31) of the MpBCs had no TILs or low (<10%) TILs, 26% (8/31) had intermediate (10–49%) TILs, and only 10% (3/31) of MpBCs had high level (≥50%) of TILs. On the pre-treatment core biopsy, 30 (68%) cases were triple-negative, 12 (27%) were hormone receptor-positive and HER2 negative, including 5 (11%) cases with low (1–10%) hormone receptor expression, and 2 (5%) were HER2 positive. Both HER2 positive cases were matrix-producing metaplastic carcinoma, HER2 equivocal by immunohistochemistry, and HER2 amplification detected by fluorescence in situ hybridization.

**Table 1:** Clinicopathological characteristics pre-NAC.

Age, median (range), years	48 (26–77)
Characteristic	*n* (%)
*BRCA* germline mutations	
Negative	28 (64%)
*BRCA1*	2 (5%)
*BRCA2*	1 (2%)
Not tested	13 (30%)
*cT stage at presentation*	
cT1	0
cT2	27 (61%)
cT3	13 (30%)
cT4	4 (9%)
*cN stage at presentation*	
cN0	28 (64%)
cN1	13 (30%)
cN2	2 (5%)
cN3	1 (2%)
*Clinical stage*	
I	1 (2%)
II	28 (61%)
III	10 (26%)
Other^a^	5 (11%)
*Tumor type*	
Pure metaplastic carcinoma	17 (39%)
Mixed metaplastic and NST	27 (61%)
*Metaplastic histologic subtype*	
Matrix-producing	19 (43%)
Squamous cell carcinoma	12 (27%)
Spindle cell carcinoma	6 (14%)
With mixed metaplastic elements	6 (14%)
No data	1 (2%)
*Tumor grade*	
I	0 (0%)
II	7 (16%)
III	31 (70%)
No data	6 (14%)
*TILs*	
<10%	20 (45%)
10–49%	8 (18%)
≥50%	3 (7%)
No data	13 (30%)
*Receptor status*	
HR−/HER2−	30 (68%)
HR+(1–10%)/HER2−	5 (11%)
HR+(>10%)/HER2−	7 (16%)
HR−/HER2+	1 (2%)
HR+(1–10%)/HER2+	1 (2%)
*Neoadjuvant chemotherapy*	
ACT	32 (73%)
ACT + platinum	9 (20%)
ACT-HP	2 (5%)
Other^b^	1 (2%)

*HR* hormonal receptor, *NST* no special type, *TILs* tumor-infiltrating lymphocytes, *ACT* doxorubicin, cyclophosphamide and taxol, *ACT-HP* doxorubicin, cyclophosphamide and taxol, plus trastuzumab and pertuzumab.

^a^Four patients had history of ipsilateral breast carcinoma, s/p breast conserving surgery, with ipsilateral breast tumor recurrence, one patient with stage IV disease at presentation.

^b^Taxol/carboplatin, then carboplatin/gemcitabine/pembrolizumab.

### Chemotherapy, clinical, and radiological response and surgical treatment

All patients received doxorubicin, cyclophosphamide, and taxol (ACT)-based NAC regimens, including 32 (73%) patients who received ACT alone, 9 (20%) with added platinum (carboplatin *n* = 8, cisplatin *n* = 1). One patient had disease progression while on taxol and carboplatin and switched to carboplatin, gemcitabine, and pembrolizumab. The two (5%) patients with *HER2* amplified MpBC received dual-anti-HER2 treatment with trastuzumab (H) and pertuzumab (P) plus chemotherapy (ACT-HP).

Clinico-radiologic response data were available for 41 patients (Table 2). Nearly half (49%, 20/41) of the patients had either no clinical or radiological response (22%, 9/41) or disease progression (27%, 11/41) while on NAC, and 51% (21/41) of patients had a clinical or radiological response. Tumors with radiologic progression had a median increase of 2.4 cm (range 1.1–12.6 cm) in the greatest dimension, while those with radiologic response had a median decrease of 1.8 cm (range: 0.3–7.7 cm) in the greatest dimension. Advanced clinical T stage (cT4) at presentation was associated with disease progression while on NAC. A significantly higher rate of clinico-radiologic response was seen in matrix-producing MpBC (78%, 14/18), compared to 27% (6/22) in non-matrix-producing MpBCs (*p* = 0.0036) (Table 2). Clinico-radiologic response was observed in 25% (3/12) squamous cell carcinoma, 40% (2/5) spindle cell carcinoma and 20% (1/5) MpBC with mixed metaplastic components. The clinicopathologic response was also more frequent among MpBCs with intermediate-to-high levels of TILs (8/11, 73%) compared to MpBCs with no or low TILs (9/18, 50%), but the difference did not reach statistical significance (*p* = 0.2732).

**Table 2:** Clinicopathological characteristics in 41 patients with clinical and radiological response assessment.

	Complete or partial response *n* = 21	No response *n* = 9	Progression *n* = 11	*p* value
Mean age, years	50	48	51	0.8159
*Clinical stage*				
I	0	1 (11%)	0	1
II	14 (67%)	7 (78%)	4 (36%)	
III	5 (24%)	0	5 (45%)	
Other	2 (10%)	1 (11%)	2 (18%)	
*Tumor type*				
Pure MpBC	8 (38%)	4 (44%)	5 (45%)	0.7557
Mixed MpBC/NST	13 (62%)	5 (56%)	6 (55%)	
*Metaplastic histologic subtype*				
MpBC—MP	14 (67%)	2 (22%)	2 (18%)	0.0036
MpBC—SCC	3 (14%)	3 (33%)	6 (55%)	
MpBC—SPC	2 (10%)	1 (11%)	2 (18%)	
MpBC—mixed	1 (5%)	3 (33%)	1 (9%)	
No data	1 (5%)	0	0	
*Tumor grade*				
II	6 (29%)	0	1 (9%)	0.0918
III	13 (62%)	8 (89%)	8 (73%)	
No data	2 (10%)	1 (11%)	2 (18%)	
*TILs*				
<10%	9 (43%)	6 (67%)	3 (27%)	0.2732
10–49%	6 (29%)	0	2 (18%)	
≥50%	2 (10%)	0	1 (9%)	
No data	4 (19%)	3 (33%)	5 (45%)	

*MpBC* metaplstic breast carcinoma, *MP* matrix producing, *NST* no special type, *SCC* squamous cell carcinoma, *SPC* spindle cell carcinoma, *TILs* tumor-infiltrating lymphocytes.

**Fig. 1:**
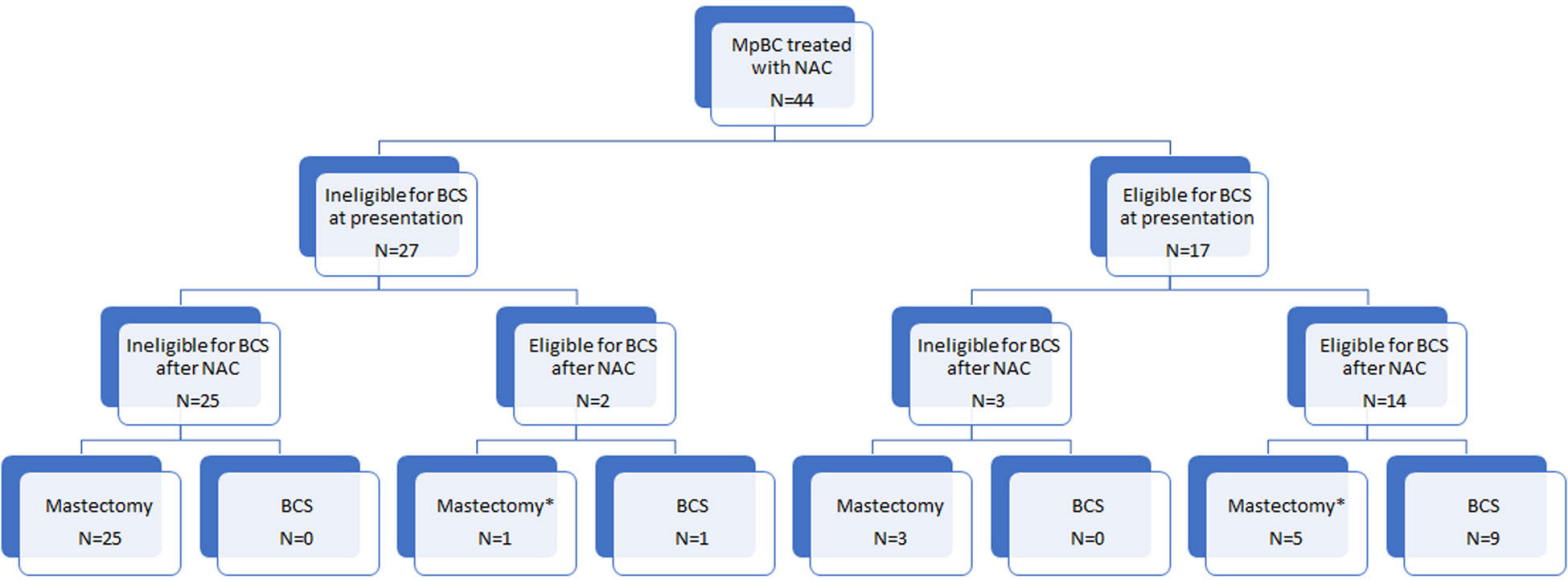
Flow chart of surgical treatment. MpBC metaplastic breast cancer, NAC neoadjuvant chemotherapy, BCS breast conversing surgery. *Patient’s choice.

Most patients (77%, 34/44) underwent mastectomy post-NAC, including 5 patients eligible for breast-conserving surgery (BCS) who opted for a mastectomy, and 23% (10/44) had successful BCS (Fig. 1). Two patients initially ineligible for BCS were downstaged to eligibility for BCS post-NAC, whereas three patients potentially eligible for BCS became ineligible due to disease progression during treatment (Fig. 1). Twenty-two (50%) patients had sentinel lymph node biopsy (SLNBx) only, 6 (14%) patients underwent SLNBx and axillary lymph node dissection (ALND), and 16 (36%) patients had ALND without SLNBx. Among the 16 patients with node-positive disease at presentation, 5 had SLNBx and intraoperative evaluation after NAC, of which, 3 (19%) patients had successful axillary downstaging and ALND was avoided.

### Post-NAC pathologic evaluation

**Fig. 2:**
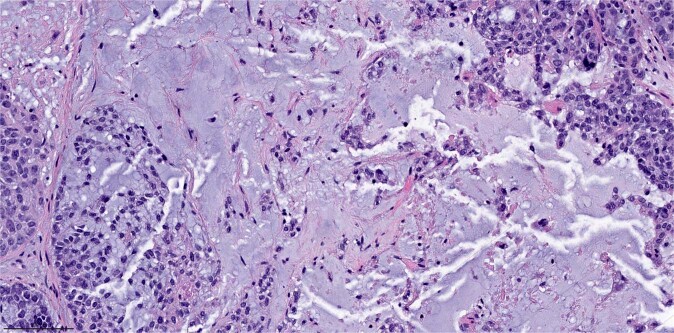
Representative micrograph of the pre-neoadjuvant treatment core biopsy of the sole patient who had pathologic complete response. Note that the core biopsy revealed a matrix-producing metaplastic carcinoma, poorly differentiated. Magnification ×200.

Only one patient had pCR, an overall pCR rate of 2% (1/44) in this cohort (Table 3). The patient was a 32 years old woman with germline *BRCA1* del exons 23–24, presented with a 3 cm mass in the right breast at 32 weeks gestation. Core biopsy revealed a matrix-producing metaplastic carcinoma (Fig. 2), poorly differentiated and of triple-negative phenotype, clinical stage II (cT2N0). The patient started NAC with ACT after a c-section. Post-NAC bilateral mastectomy and sentinel lymph node biopsy revealed no residual invasive carcinoma. She remained without evidence of disease at the last follow-up (53 months).

**Table 3:** Rates of pathologic complete response in breast and lymph node stratified by histologic subtype of metaplastic carcinoma.

Metaplastic histologic subtype	Overall pCR (ypT0N0) *n* (%)	Breast pCR (ypT0) *n* (%)	Total ypN0 *n* (%)	Nodal pCR in cN +*n* (%)
All cases	1/44 (2%)	2/44 (5%)	26/44 (59%)	3/16 (19%)
Matrix-producing	1/19 (5%)	2/19 (11%)	13/19 (68%)	2/7 (29%)
Squamous cell carcinoma	0/12	0/12	5/12 (42%)	1/6 (17%)
Spindle cell carcinoma	0/6	0/6	5/6 (83%)	0/0
With mixed metaplastic elements	0/6	0/6	2/6 (33%)	0/3
No data	0/1	0/1	1/1 (100%)	0/0

One patient had a pCR in the breast but had residual metastatic disease in one lymph node (Table 3). Among the 16 patients with biopsy-proven nodal involvement pre-NAC, 3 (19%) patients had nodal pCR post-NAC (Table 3).

The median residual pathologic tumor size was 2.5 cm (range: a few cells—24.8 cm). Eighteen (41%) patients had axillary lymph node involvement post NAC with a median number of 2 (range 1–16) positive lymph nodes. Sixteen (36%) patients had macrometastases, one (2%) patient had micrometastasis, and one (2%) patient had isolated tumor cells. The median tumor bed area was 9.6 cm^2^ (range: 1.7–81.0 cm^2^). Among the 22 patients with slides available for residual cancer cellularity assessment, the median overall cancer cellularity was 55% (range: 0–95%). RCB was assessed in 21 patients with residual tumor, one (5%) patient was RCB class I, 17 (81%) patients were class II, and 3 (14%) patients were class III.

Receptor status post-NAC was available in 38 cases, all were triple-negative (34/38, 89%) or hormonal receptor low (1–10%) positive and HER2 negative (4/38, 11%). Receptor status between pre- and post-NAC was concordant in 66% (25/38) and discordant in 34% (13/38) of patients. The most common receptor conversion was from hormonal receptor low positive (1–10%) to negative (5/38, 13%), followed by hormonal receptor-positive to negative (4/38, 11%), positive to low positive (2/38, 5%), and negative to low positive (2/38, 5%). Targeted sequencing by MSK-IMPACT^29,30^ was performed in 7 patients, on pre-NAC samples in 2 patients, post-NAC in 2 patients, pre- and post-NAC in 1 patient, and distant metastasis in 2 patients. Recurrent somatic mutations observed included *TP53* (7/7), *PTEN* (3/7), *PIK3R1* (2/7), and *SOX17* (2/7). Somatic mutations affecting PI3K pathway genes (*PTEN, PIK3R1, INPP4B*) were detected in 71% (5/7) of cases.

### Outcome analysis

Follow-up information was available for 39 patients, with a median follow-up of 34.9 months (range: 1.6–140.4). Thirteen patients developed distant metastases. The 3-year disease-free survival and overall survivals were 58% and 65%, respectively. Residual cancer burden (RCB) was the only factor associated with disease-free survival by log-rank test (*p* = 0.0001) (Table 4). On univariate analysis, post-NAC tumor size ≤ 2 cm (*p* = 0.046), post-NAC nodal status (*p* = 0.037), and RCB (*p* = 0.0007) were associated with overall survival (Table 4). Multivariate analysis was not performed due to the small sample size and missing data for RCB in a subset of patients.

**Table 4:** Clinicopathologic factors associated with disease-free survival and overall survival.

Characteristics	*p* value	
	Disease-free survival	Overall survival
Clinicoradiological response	0.79	1
Metaplastic histologic subtype	0.49	0.77
Post-treatment tumor size (ypT0–1 vs. ypT2–3)	0.29	0.046
Post-treatment nodal status (ypN0 vs. ypN1–3)	0.064	0.034
RCB class (I–III)	0.001	0.0007
Pre-treatment tumor grade	0.14	0.11
Post-treatment tumor grade	0.26	0.37
Pre-treatment receptor status	0.81	0.72
Post-treatment receptor status	0.31	0.3

## Discussion

MpBC is usually of triple-negative phenotype and appears more aggressive than conventional TNBC. The National Comprehensive Cancer Network (NCCN) clinical practice guidelines do not have specific treatment recommendations for MpBC distinct from that for invasive breast carcinoma in general^31^. NAC has become standard practice for patients with clinical stage II–III TNBCs. However, in this study, we observed a poor response to NAC in patients with MpBC, with only one patient achieving pCR, 22% of the patients exhibited no clinical and radiological response, and 27% with progression while on NAC, in contrast to the 30–50% pCR rate in TNBC reported in the literature^20–23^.

Benefits of NAC include downstaging the primary tumor to allow BCS and downstaging the axilla to avoid axillary dissection in patients presenting with node-positive disease. In our study, only two patients were sufficiently downstaged from ineligible to eligible for BCS post-NAC, and three patients progressed from eligible to ineligible for BCS during treatment.

Prior studies from our institution demonstrated nodal pCR rates of 41–48% in patients with cT1–3 biopsy-proven N1 breast cancer treated with NAC^32,33^. Similarly, in the ACOSOG Z1071 trial, of the 694 patients with biopsy-proven node-positive breast cancer (cT0–4 N1–2 M0), the nodal pCR rate was 41%^34^. The nodal pCR rate in patients with TNBC in the ACOSOG Z1071 trial was 49% (84/170)^34^. In contrast, in the current study, among 16 MpBC patients with clinically node-positive disease at presentation, only 19% (3/16) had nodal pCR, a significantly lower rate than that in patients with TNBC in the ACOSOG Z1071 trial (*p* = 0.0199).

Several prior studies have reported responses to NAC in MpBC. Han et al. reported a 17% pCR rate among 29 patients with MpBC treated with NAC^25^. Al-Hilli et al. reported an 11% pCR rate in 18 patients with MpBC^26^. Cimino-Mathews et al. reported six MpBC patients treated with NAC, with one patient (16%) achieved pCR^24^. The differences in rates of pCR across published studies may be due to the very small sample size in some and the heterogeneous morphology of MpBC. There are no consensus criteria that differentiate metaplastic carcinoma from invasive ductal carcinoma with metaplastic features^1^. Lack of consensus inclusion criteria among studies may contribute to sample heterogeneity and differences in pCR rates. In the study by Han et al., any case showing an unequivocal metaplastic tumor component was included in the study^25^. Of the 29 patients with MpBC treated with NAC, 15 (52%) had mixed metaplastic carcinoma and invasive carcinoma NST, including 9 (31%) patients in which the invasive carcinoma NST was the predominant component^25^. Similarly, the study by Cimino-Mathews et al. included cases with any degree of metaplastic element^24^. Whereas in our study, we included only cases with predominant metaplastic component, following the WHO classification guidelines^1^, potentially accounting for the lower response rates we observed.

Although the overall response rates in our study were low, differences in response based on histology were observed. The matrix-producing subtype was significantly associated with clinical-radiologic response. Of the 18 patients with matrix-producing subtype and with clinic-radiologic response assessment, 14 (78%) patients had a response to NAC, compared to 27% in non-matrix-producing MpBCs (*p* = 0.0036). The only patient with pCR had a matrix-producing MpBC. Han et al. also reported that matrix-producing subtype was significantly associated with pCR in their study cohort^25^.

The presence of tumor-infiltrating lymphocytes (TILs) is an independent predictor of response to NAC^35,36^. Denkert et al. reported a pCR rate of over 40% in lymphocyte-predominant breast cancer (LPBC), defined as breast cancer with more than 60% of TILs^35^. TNBC has the highest incidence of LPBC^36,37^. In a systematic review, a median of 20% (range 4–37%, *n* = 1620 patients) of patients with TNBC demonstrated LPBC, defined as at least 50% or 60% TILs^37^. MpBCs, however, are less frequently associated with high levels of TILs. In one study, a high level of TILs, defined as ≥60% of TILs, was observed in 33% of squamous cell carcinoma but none of the matrix-producing MpBC or spindle cell carcinomas^38^. In our study, high levels of TILs (≥50%) were only seen in 10% (3/31) of MpBCs, including 2 of 10 squamous cell carcinomas and 1 of 13 matrix-producing MpBC with TILs assessment in pre-NAC core biopsy.

Whole-exome sequencing of MpBCs demonstrated a repertoire of somatic mutations distinct from that of TNBC NST^39^. Although both MpBC and TNBC NST harbor frequent *TP53* mutations at similar rates, MpBCs more frequently harbored mutations in *PIK3CA* (29%), *PIK3R1* (11%), *ARID1A* (11%), *FAT1* (11%), and *PTEN* (11%) in comparison to TNBC NST^39^. When compared to TNBC NST, MpBCs were more frequently associated with mutations in the PI3K/AKT/mTOR pathway (57% vs. 22%) and the Wnt pathway (51% vs. 28%)^39^. The difference in somatic mutations may contribute to the poor clinical outcomes in MpBC. A sequencing analysis in the neoadjuvant GeparSepto trial showed a significantly reduced pCR rate in *PIK3CA*-mutated breast cancer compared with *PIK3CA* wild-type breast cancer (23% vs. 38.8%, *p* < 0.0001)^40^. In our study cohort, sequencing analysis was only performed in a subset of patients (*n* = 7), but among these patients, 71% (5/7) had somatic mutations affecting genes in the PI3K pathway.

Our study has several limitations. It is a retrospective study with the possibility of selection bias. Due to the rarity of MpBC, our sample size is limited. Data analysis was further restricted to patients with complete data in the report or with slides retrievable for review. Despite these limitations, our study is the largest series of MpBC treated with NAC with detailed clinicopathologic annotation.

In conclusion, MpBC had a poor response to conventional NAC. Only one (2%) patient achieved a pathological complete response in our cohort, and 27% had disease progression during NAC. The poor response to NAC in patients with MpBC raises the question of the utility of conventional NAC for these patients. While overall a low rate of clinicoradiologic and pathologic response, variation in response to NAC exists based on the histologic subtype, with some clinical benefit from NAC observed in matrix-producing MpBC, illustrating the heterogeneity within MpBC.

## Methods

### Patient cohort

Patients with MpBC treated with NAC at our center from 2002 to 2018 were retrospectively reviewed. Clinical and radiological features were obtained from the electronic medical record. Clinico-radiologic response was determined by a change in size between pre-NAC and post-NAC on physical examination and/or radiological imaging by the same imaging modality. Clinical lymph node status was determined by physical examination and imaging study, confirmed with fine-needle aspiration or core needle biopsy prior to NAC. Clinical follow-up post-surgical treatment and recurrence events were recorded, and disease-free survival and overall survival were evaluated. This study was approved by the institutional review board of Memorial Sloan Kettering Cancer Center (protocol #17-287). A waiver of consent was granted by the institutional ethics committee because this work involves no more than minimal risk to the participants or their privacy.

### Pathologic evaluation

Pathologic characteristics were retrieved from the pathology report and central slide review. Available diagnostic slides were reviewed by two breast pathologists (W.W. and H.W.) to confirm diagnoses and histologic features. A diagnosis of MpBC was rendered if metaplastic features were predominant in the tumor. The extent of TILs was assessed on pre-NAC biopsy according to the recommendations by the international TILs working group^41^. Pathologic evaluation post-NAC followed the recommendations of the international working group^42^. Pathological complete response (pCR) was defined as no evidence of invasive disease in the breast and lymph nodes, with or without residual ductal carcinoma in situ (ypT0/pTis ypN0). RCB was determined from the primary tumor bed area, overall cancer cellularity, percentage of cancer that is in situ disease, number of positive lymph nodes, and diameter of largest metastasis in lymph nodes, using the MD Anderson RCB calculator^43^. Estrogen receptor, progesterone receptor, and HER2 assessment followed the ASCO/CAP guidelines^44,45^ and were recorded in both the pre-NAC biopsy and the post-NAC surgical specimens. Next-generation sequencing data using MSK-IMPACT were recorded when available^29,30^.

### Statistics

Fisher’s Exact test was used to evaluate associations between categorical variables and response to therapy. Survival outcomes were analyzed with the Kaplan–Meier method and statistical significance were determined by log-rank test. A *p* value < 0.05 was considered statistically significant.

### Reporting summary

Further information on research design is available in the Nature Research Reporting Summary linked to this article.

## Supplementary information

Reporting Summary

## Data Availability

The data generated and analyzed during this study are described in the following data record: 10.6084/m9.figshare.14823633^46^. All data are contained in the three Excel files: ‘Clinicopathological characteristics.xlsx’, ‘Rates of pathologic complete response.xlsx’ and ‘Clinicopathologic factors associated outcomes.xlsx’. These files are housed on institutional storage and are not publicly available for the following reason: data contain information that could compromise research participant privacy and informed consent to share participant-level data was not obtained prior to or during data collection. Data will be made available to authorized researchers who have received approval from the Memorial Sloan Kettering Cancer Center Institutional Review Board. Any enquiries relating to the data should be directed to the corresponding author.

## References

[1] Reis-Filho, J. S. et al. Metaplastic carcinoma. In The WHO Classification of Tumours Breast Tumours. 5th ed. Lyon: IARC Press, 2019.

[2] Van Hoeven KH, Drudis T, Cranor ML, Erlandson RA, Rosen PP (1993). Low-grade adenosquamous carcinoma of the breast. A clinocopathologic study of 32 cases with ultrastructural analysis. Am. J. Surg. Pathol..

[3] Bataillon G (2018). High rate of PIK3CA mutations but no TP53 mutations in low-grade adenosquamous carcinoma of the breast. Histopathology.

[4] Gobbi H, Simpson JF, Borowsky A, Jensen RA, Page DL (1999). Metaplastic breast tumors with a dominant fibromatosis-like phenotype have a high risk of local recurrence. Cancer.

[5] Sneige N (2001). Low-grade (fibromatosis-like) spindle cell carcinoma of the breast. Am. J. Surg. Pathol..

[6] Schroeder MC, Rastogi P, Geyer CE, Miller LD, Thomas A (2018). Early and locally advanced metaplastic breast cancer: presentation and survival by receptor status in surveillance, epidemiology, and end results (SEER) 2010–2014. Oncologist.

[7] Langlands F (2016). Imaging overview of metaplastic carcinomas of the breast: a large study of 71 cases. Br. J. Radiol..

[8] Reis-Filho JS (2006). Metaplastic breast carcinomas are basal-like tumours. Histopathology.

[9] Rakha EA (2017). Immunoprofile of metaplastic carcinomas of the breast. Histopathology.

[10] Pezzi CM (2007). Characteristics and treatment of metaplastic breast cancer: analysis of 892 cases from the National Cancer Data Base. Ann. Surg. Oncol..

[11] Ong CT (2018). Metaplastic breast cancer treatment and outcomes in 2500 patients: a retrospective analysis of a National Oncology Database. Ann. Surg. Oncol..

[12] El Zein D (2017). Metaplastic carcinoma of the breast is more aggressive than triple-negative breast cancer: a study from a single institution and review of literature. Clin. Breast Cancer.

[13] Jung SY (2010). Worse prognosis of metaplastic breast cancer patients than other patients with triple-negative breast cancer. Breast Cancer Res. Treat..

[14] Hennessy BT (2005). Squamous cell carcinoma of the breast. J. Clin. Oncol..

[15] Davis WG (2005). Metaplastic sarcomatoid carcinoma of the breast with absent or minimal overt invasive carcinomatous component: a misnomer. Am. J. Surg. Pathol..

[16] Lester TR (2012). Metaplastic sarcomatoid carcinoma of the breast appears more aggressive than other triple receptor-negative breast cancers. Breast Cancer Res. Treat..

[17] Bae SY (2011). The prognoses of metaplastic breast cancer patients compared to those of triple-negative breast cancer patients. Breast Cancer Res. Treat..

[18] Okada N (2010). Metaplastic carcinoma of the breast. Hum. Pathol..

[19] Nelson RA, Guye ML, Luu T, Lai LL (2015). Survival outcomes of metaplastic breast cancer patients: results from a US population-based analysis. Ann. Surg. Oncol..

[20] Cortazar P (2014). Pathological complete response and long-term clinical benefit in breast cancer: the CTNeoBC pooled analysis. Lancet.

[21] von Minckwitz G (2012). Definition and impact of pathologic complete response on prognosis after neoadjuvant chemotherapy in various intrinsic breast cancer subtypes. J. Clin. Oncol..

[22] Schmid P (2020). Pembrolizumab for early triple-negative breast cancer. N. Engl. J. Med..

[23] von Minckwitz G (2014). Neoadjuvant carboplatin in patients with triple-negative and HER2-positive early breast cancer (GeparSixto; GBG 66): a randomised phase 2 trial. Lancet Oncol..

[24] Cimino-Mathews A (2016). A clinicopathologic analysis of 45 patients with metaplastic breast carcinoma. Am. J. Clin. Pathol..

[25] Han M (2019). Metaplastic breast carcinoma: a clinical-pathologic study of 97 cases with subset analysis of response to neoadjuvant chemotherapy. Mod. Pathol..

[26] Al-Hilli Z (2019). Metaplastic breast cancer has a poor response to neoadjuvant systemic therapy. Breast Cancer Res. Treat..

[27] Corso G (2021). Metaplastic breast cancer: prognostic and therapeutic considerations. J. Surg. Oncol..

[28] Tadros, A. B. et al. Survival outcomes for metaplastic breast cancer differ by histologic subtype. *Ann. Surg. Oncol*. Epub 2021 Jan 2. 10.1245/s10434-020-09430-5 (2021). 10.1245/s10434-020-09430-5 33389291

[29] Cheng DT (2015). Memorial Sloan Kettering-Integrated Mutation Profiling of Actionable Cancer Targets (MSK-IMPACT): a hybridization capture-based next-generation sequencing clinical assay for solid tumor molecular oncology. J. Mol. Diagn..

[30] Zehir A (2017). Mutational landscape of metastatic cancer revealed from prospective clinical sequencing of 10,000 patients. Nat. Med..

[31] NCCN Clinical Practice Guidelines in Oncology (NCCN Guidelines®)—Breast Cancer. https://www.nccn.org. 2021 (version 1. 2021).

[32] Mamtani A (2016). How often does neoadjuvant chemotherapy avoid axillary dissection in patients with histologically confirmed nodal metastases? Results of a Prospective Study. Ann. Surg. Oncol..

[33] Montagna G (2020). Selecting node-positive patients for axillary downstaging with neoadjuvant chemotherapy. Ann. Surg. Oncol..

[34] Boughey JC (2014). Tumor biology correlates with rates of breast-conserving surgery and pathologic complete response after neoadjuvant chemotherapy for breast cancer: findings from the ACOSOG Z1071 (Alliance) Prospective Multicenter Clinical Trial. Ann. Surg..

[35] Denkert C (2010). Tumor-associated lymphocytes as an independent predictor of response to neoadjuvant chemotherapy in breast cancer. J. Clin. Oncol..

[36] Denkert C (2018). Tumour-infiltrating lymphocytes and prognosis in different subtypes of breast cancer: a pooled analysis of 3771 patients treated with neoadjuvant therapy. Lancet Oncol..

[37] Stanton SE, Adams S, Disis ML (2016). Variation in the incidence and magnitude of tumor-infiltrating lymphocytes in breast cancer subtypes: a systematic review. JAMA Oncol..

[38] Lien HC (2021). Tumor-infiltrating lymphocyte abundance and programmed death-ligand 1 expression in metaplastic breast carcinoma: implications for distinct immune microenvironments in different metaplastic components. Virchows Arch.

[39] Ng CKY (2017). The landscape of somatic genetic alterations in metaplastic breast carcinomas. Clin. Cancer Res..

[40] Loibl S (2019). Mutational diversity and therapy response in breast cancer: a sequencing analysis in the neoadjuvant geparsepto trial. Clin. Cancer Res..

[41] Salgado R (2015). The evaluation of tumor-infiltrating lymphocytes (TILs) in breast cancer: recommendations by an International TILs Working Group 2014. Ann. Oncol..

[42] Provenzano E (2015). Standardization of pathologic evaluation and reporting of postneoadjuvant specimens in clinical trials of breast cancer: recommendations from an international working group. Mod. Pathol..

[43] Symmans WF (2017). Long-term prognostic risk after neoadjuvant chemotherapy associated with residual cancer burden and breast cancer subtype. J. Clin. Oncol..

[44] Allison KH (2020). Estrogen and progesterone receptor testing in breast cancer: ASCO/CAP guideline update. J. Clin. Oncol..

[45] Wolff AC (2018). Human epidermal growth factor receptor 2 testing in breast cancer: American Society of Clinical Oncology/College of American Pathologists Clinical Practice Guideline Focused Update. J. Clin. Oncol..

[46] Wong, W. Metadata record for the article: poor response to neoadjuvant chemotherapy in metaplastic breast carcinoma. figshare 10.6084/m9.figshare.14823633 (2021). 10.1038/s41523-021-00302-z PMC8298632 34294707

